# The inflammatory milieu within the pancreatic cancer microenvironment correlates with clinicopathologic parameters, chemoresistance and survival

**DOI:** 10.1186/s12885-015-1820-x

**Published:** 2015-10-24

**Authors:** Daniel Delitto, Brian S. Black, Heather L. Sorenson, Andrea E. Knowlton, Ryan M. Thomas, George A. Sarosi, Lyle L. Moldawer, Kevin E. Behrns, Chen Liu, Thomas J. George, Jose G. Trevino, Shannon M. Wallet, Steven J. Hughes

**Affiliations:** 1 Department of Surgery, College of Medicine, University of Florida Health Science Center, Room 6116, Shands Hospital, 1600 SW Archer Rd, Gainesville, FL 32610 USA; 2 Department of Oral Biology, College of Dentistry, University of Florida Health Science Center, Gainesville, FL 32610 USA; 3 North Florida/South Georgia Veterans Health System, Department of Surgery, University of Florida College of Medicine, Gainesville, FL 32610 USA; 4 Department of Pathology, College of Medicine, University of Florida Health Science Center, Gainesville, FL 32610 USA; 5 Department of Medicine, College of Medicine, University of Florida Health Science Center, Gainesville, FL 32610 USA

**Keywords:** Inflammation, Cytokines, Chemokines, Growth factors, Pancreatic cancer, Tumor microenvironment

## Abstract

**Background:**

The tumor microenvironment impacts pancreatic cancer (PC) development, progression and metastasis. How intratumoral inflammatory mediators modulate this biology remains poorly understood. We hypothesized that the inflammatory milieu within the PC microenvironment would correlate with clinicopathologic findings and survival.

**Methods:**

Pancreatic specimens from normal pancreas (*n* = 6), chronic pancreatitis (*n* = 9) and pancreatic adenocarcinoma (*n* = 36) were homogenized immediately upon resection. Homogenates were subjected to multiplex analysis of 41 inflammatory mediators.

**Results:**

Twenty-three mediators were significantly elevated in adenocarcinoma specimens compared to nonmalignant controls. Increased intratumoral IL-8 concentrations associated with larger tumors (*P* = .045) and poor differentiation (*P* = .038); the administration of neoadjuvant chemotherapy associated with reduced IL-8 concentrations (*P* = .003). Neoadjuvant therapy was also associated with elevated concentrations of Flt-3 L (*P* = .005). Elevated levels of pro-inflammatory cytokines IL-1β (*P* = .017) and TNFα (*P* = .033) were associated with a poor histopathologic response to neoadjuvant therapy. Elevated concentrations of G-CSF (*P* = .016) and PDGF-AA (*P* = .012) correlated with reduced overall survival. Conversely, elevated concentrations of FGF-2 (*P* = .038), TNFα (*P* = .031) and MIP-1α (*P* = .036) were associated with prolonged survival.

**Conclusion:**

The pancreatic cancer microenvironment harbors a unique inflammatory milieu with potential diagnostic and prognostic value.

## Background

Pancreatic adenocarcinoma (PC) is the fourth leading cause of cancer deaths in the United States, due in part to nearly universal resistance to cytotoxic chemotherapy. Gemcitabine-based therapies achieve clinical benefit in approximately 24 % of patients with PC [1], but the overall survival advantages are sobering, ranging from a few weeks to months [1–3]. Complete surgical resection offers patients with PC the greatest survival benefit. However, this is achievable in fewer than 20 % of patients presenting with PC [4]. As a result, PC is projected to be the second leading cause of cancer deaths by 2030 [5]. There is a tremendous need to discover novel biomarker (s) or panels of biomarkers that can aid in detecting PC earlier, improving prognostic evaluation and predicting response to chemotherapy.

Inflammation within the PC microenvironment has been mechanistically linked to tumor progression and chemoresistance through NF-κB, IL-6, toll-like receptor and TGF-β signaling pathways [6–10]. However, the diagnostic and prognostic value of the inflammatory milieu within the PC microenvironment remains essentially undefined. While survival gains from immune cell infiltration into the tumor microenvironment have been conclusively demonstrated in colorectal and ovarian cancer [11–13], similar investigations have not yielded consistent results in PC [14, 15]. Patients with chronic pancreatitis are 5–15 times more likely to develop PC [16] and insights into the association between inflammation and PC stems from investigations of chronic pancreatitis. Potential environmental sequelae of pancreatitis such as hypoxia, the presence of reactive oxygen species, and acidosis may influence the development of PC [17]. Additionally, numerous soluble mediators, including TNF-α [18], TGF-α [19], TGF-β [20], IL-1β [21], IL-1α [22], IL-6 [23, 24], IL-8 [25], VEGF [26], and others have been implicated in PC carcinogenesis, tumor progression, and treatment resistance. However, the relationship between the inflammatory milieu and the spectrum of disease from normal pancreas to pancreatitis to pancreatic cancer has not yet been characterized. Therefore, the translational relevance of the microenvironmental inflammatory milieu to PC development and progression remains speculative.

We examined the inflammatory milieu present in the PC microenvironment from 36 freshly resected tumor specimens using a forty-one-item panel of cytokines, chemokines and growth factors to test the hypothesis that expression levels of these mediators harbor diagnostic and prognostic value. We first compared the inflammatory milieu of PC to that of pancreatitis (*n* = 9) and normal pancreas (*n* = 6). Inflammatory mediators were further evaluated in relation to prognostic clinicopathologic parameters, administration of neoadjuvant therapy, treatment resistance and patient survival. These data bring the field one step closer to the identification of biomarker panels that can aid in detecting disease earlier and classifying patients with respect to response to chemotherapy and most importantly, prognosis.

## Materials and methods

### Patient cohorts

A prospectively maintained database approved by the Institutional Review Board at the University of Florida (353–2007) was utilized for sample selection. Written informed consent was obtained from all participants. In total, 51 samples were included in this study. Using pathologically verified diagnoses, samples were placed into one of three experimental groups: normal pancreas (*n* = 6), chronic pancreatitis (*n* = 9) and pancreatic carcinoma (*n* = 36). Indications for resection of ‘normal’ pancreata included duodenal adenomas (n = 3), remotely located neuroendocrine tumors (*n* = 2) and a ductal squamoid cyst (*n* = 1). Of the 36 patients with pathologically confirmed pancreatic adenocarcinoma, all underwent resection with curative intent, 10 whom completed gemcitabine/abraxane-based neoadjuvant chemotherapy. Pathologic response to neoadjuvant chemotherapy was graded by clinical pathologists upon resection using a validated scale [27]. Briefly, histopathologic response to neoadjuvant therapy was broadly grouped into complete (>90 % of tumor cells destroyed), moderate (10–90 % of tumor cells destroyed) and poor (<10 % of tumor cells destroyed). All 36 patients had at least 6 months of clinical follow-up for survival analysis.

### Pancreatic tissue harvest

Resected pancreatic tissue was immediately weighed and placed in cell lysis buffer (Cell Signaling Technologies, Danvers, MA) with a protease inhibitor cocktail (Sigma-Aldrich, St. Louis, MO). Immediately adjacent tissues were preserved in formalin for histologic verification of pathology. Tissues were dissociated mechanically and further homogenized using the FastPrep-24 system according to the manufacturer’s protocol (MP Biomedicals, Santa Ana, CA). Homogenates were stored at −80 °C until soluble mediator analysis could be performed.

### Soluble mediator analysis

Homogenates were then probed for soluble mediators using the Milliplex® Premixed 41-Plex Immunology Multiplex Assay (Merck Millipore, Darmstadt, Germany) according to the manufacturer’s protocol. Specifically, supernatants from tissue homogenates were incubated in filter bottom microtiter plates (EMD Millipore, San Jose, CA) with beads coated with primary antibodies overnight at 4C. After washing, PE- conjugated anti-cytokine antibodies were added and incubated for additional 2 h at room temperature. Following washing, data was acquired on a Luminex 200 (EMD Millipore, San Jose, CA) and analyzed with Milliplex Software (EMD Millipore, San Jose, CA). Concentrations were quantified using a standard curve and 5 parameter logistics to determine pg/mL concentrations.

All cytokine concentrations were normalized to total protein concentrations using detergent compatible protein quantification (Bio-Rad, Hercules, CA). Soluble mediator concentrations were then converted to pg/mg of tissue as follows: pg/ml divided by mg/ml of total protein.

### Statistical analysis

All statistical analysis was performed using SPSS version 22.0 (IBM SPSS Statistics for Windows; IBM Corp). For each normalized tissue cytokine concentration, represented in picograms per milligram of total protein (pg/mg protein), normality was assessed using the Shapiro-Wilk test. Since all normalized cytokine concentrations did not display normal distributions (*P* < 0.05), non-parametric testing was employed to evaluate differences. In this manner, the Mann Whitney U test was incorporated for binomial categorical variables, and *P* < 0.05 was considered statistically significant. Additionally, Spearman’s rank correlation coefficients were employed to determine significant associations between continuous variables. Overall survival was calculated using the following formula: Number of days from date of surgery to death or the date of last follow-up, whichever came first, divided by 365.25 (accounting for leap years), multiplied by 12 to obtain the time in months. Kaplan-Meier survival curves were generated using median intratumoral concentration to dichotomize PC specimens into cytokine_high_ and cytokine_low_ groups. The log-rank (Mantel-Cox) test was used to evaluate statistical significance. Additionally, a univariate Cox proportional hazards model was used to generate hazard ratios. Each soluble mediator was then incorporated into a multivariate proportional hazards model with the degree of lymphatic metastasis, as this was the only clinicopathologic parameter demonstrating a significant correlation with survival (*P* < .05) on univariate analysis.

## Results

### Pancreatic adenocarcinoma has a distinct intratumoral inflammatory milieu

Establishing the diagnosis of PC remains a significant clinical problem that delays initiation of therapy, impacts enrollment in clinical trials, and mandates that patients undergo major surgical procedures in the absence of definitive findings. In order to determine whether the intratumoral inflammatory milieu may have diagnostic value, we measured the concentrations of 41 cytokines, chemokines and growth factors in 51 freshly homogenized pancreatic surgical samples. We found no significant differences in any of the normalized cytokine concentrations when comparing normal pancreatic tissue (*n* = 6) to that of chronic pancreatitis (*n* = 9). Thus, pairwise comparisons between nonmalignant tissue (*n* = 15) and adenocarcinoma (*n* = 36) as well as between pancreatitis alone (*n* = 9) and adenocarcinoma (*n* = 36) were performed (Table 1). Of the 41-protein-panel of cytokines, chemokines and growth factors evaluated, the concentrations of 23 emerged as significantly higher in pancreatic cancer compared to nonmalignant tissue. The most significant differences (*P* < .001) were observed for Eotaxin, IP-10, MCP1, MCP3, MDC, IL-1α, IL-1RA, IL-7, and IL-8. Interestingly, all but 3 (FGF-2, RANTES and IL1β) of the 23 mediators that emerged as significant when comparing pancreatic cancer to nonmalignant tissue also emerged as significant when comparing pancreatic cancer to pancreatitis. Together these data suggest that pancreatic adenocarcinoma has a distinct inflammatory milieu when compared to that of nonmalignant pancreatic tissues, including that of chronic pancreatitis.

**Table 1:** Inflammatory milieu within pancreatic tissue is predictive of malignancy

	Normal pancreas (*n* = 6)	Pancreatitis (*n* = 9)	Pancreatic cancer (*n* = 36)	*P* value nonmalignant vs. Pancreatic cancer	*P* value pancreatitis vs. Pancreatic cancer
Growth factors					
FGF-2	761 (221)	951 (187)	1674 (177)	.009	.063
PDGF-BB	117 (46)	102 (48)	244 (63)	.026	.022
VEGF	151 (46)	76 (20)	252 (59)	.119	.046
Chemokines					
Eotaxin	21.8 (12.8)	21.4 (12.9)	90.4 (16.3)	<.001	.002
Fractalkine	55.1 (12.8)	43.0 (9.4)	71.1 (3.6)	.010	.009
Gro	163 (67)	463 (296)	531 (87)	.007	.043
IP-10	69 (33)	70 (32)	620 (148)	<.001	<.001
MCP1	437 (136)	378 (104)	1615 (302)	<.001	<.001
MCP3	4.7 (1.9)	2.5 (0.8)	16.6 (2.8)	<.001	<.001
MDC	27 (11)	70 (36)	143 (17)	<.001	.009
MIP-1α	14.3 (4.9)	16.9 (7.6)	37.9 (4.6)	.001	.009
MIP-1β	22.1 (9.4)	14.3 (5.0)	38.4 (6.1)	.007	.008
RANTES	766 (261)	1066 (228)	1929 (235)	.032	.178
Cytokines					
GM-CSF	4.1 (2.0)	1.4 (0.3)	14.0 (3.9)	<.001	<.001
IFNα2	13.0 (2.7)	8.7 (2.3)	19.9 (2.4)	.013	.012
IL-1α	1.8 (0.5)	3.2 (0.6)	12.2 (2.0)	<.001	.003
IL-1RA	157 (144)	46 (21)	500 (96)	<.001	<.001
IL-1β	0.6 (0.1)	0.9 (0.1)	1.3 (0.2)	.032	.262
IL-6	13.3 (5.3)	5.8 (2.1)	71.8 (23.3)	<.001	<.001
IL-7	3.9 (1.0)	4.5 (1.0)	10.4 (0.8)	<.001	<.001
IL-8	160 (154)	61 (37)	848 (161)	<.001	.002
IL-15	1.7 (0.2)	2.0 (0.3)	3.5 (0.4)	.001	.021
TNFα	1.6 (0.7)	1.4 (0.4)	3.3 (0.4)	.002	.010

Concentrations expressed as mean (SE) in units of pg/mg protein. All significant comparisons are shown for which *P* < 0.05 using the Mann Whitney U test

### Elements of the intratumoral inflammatory milieu strongly associate with the administration of neoadjuvant cytotoxic chemotherapy

Dynamic changes accompanying the administration of cytotoxic chemotherapy within the PC microenvironment remain poorly described. In order to determine whether differences within intratumoral inflammatory milieu associate with the administration of cytotoxic chemotherapy, patients were dichotomized into groups based on the administration of neoadjuvant therapy. Indeed, significantly lower levels of intratumoral IL-8 were observed in PC specimens from patients treated with neoadjuvant gemcitabine-based regimens compared to those from treatment naïve patients (median concentration 1129 pg/mg protein vs. 114 pg/mg protein; *P* = .003) (Table 2). Conversely, high intratumoral concentrations of Flt-3 L and IL-2 correlated with the administration of neoadjuvant chemotherapy. Together these data suggest that the administration of cytotoxic chemotherapy alters the inflammatory microenvironment in PC.

**Table 2:** Intratumoral milieu correlates with the administration of cytotoxic chemotherapy

	Neoadjuvant Chemotherapy		
	No (*n* = 26)	Yes (*n* = 10)	*P* value
Flt3L	15.9 (1.6)	24.0 (3.5)	.005
IL-1α	15.0 (2.6)	5.0 (0.9)	.006
IL-8	1129 (197)	114 (35)	.003

All significant comparisons are shown for which *P* < 0.05 using the Mann Whitney U test

**Fig. 1:**
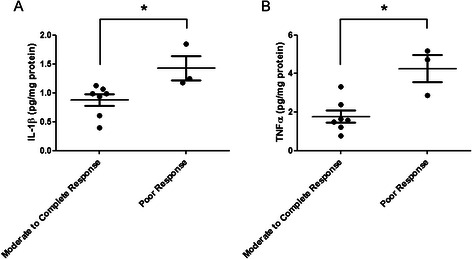
Th1-associated cytokines within the tumor microenvironment correlate with treatment resistance in PC. Distributions are displayed comparing intratumoral concentrations of **a** IL-1β and **b** TNFα in homogenates of pancreatic adenocarcinoma with histopathologic response to neoadjuvant chemotherapy. Bars represent mean values with standard error of the mean. *P < 0.05 for each association using the Mann Whitney U test

Poor histopathologic response to neoadjuvant chemotherapy appears to associate with poor clinical outcomes, although this phenomenon continues to be debated [28–30]. In order to determine if the intratumoral milieu could offer insights into the degree of clinical response to cytotoxic chemotherapy, histopathologic response to neoadjuvant chemotherapy was correlated to soluble mediator concentrations. Clinically, resected PC specimens with a poor histopathologic response to neoadjuvant therapy represent a group of treatment-resistant tumors. Indeed, significantly higher levels of the pro-inflammatory cytokines IL-1β and TNFα were observed in tumors from this population compared to tumors displaying a moderate to complete pathologic response to cytotoxic chemotherapy (Fig. 1). These preliminary data provide rationale for the continued evaluation of potential biologic mechanisms within the tumor microenvironment by which resistance to cytotoxic chemotherapy is maintained.

### Variations within the intratumoral inflammatory milieu correlate with clinicopathologic features

**Fig. 2:**
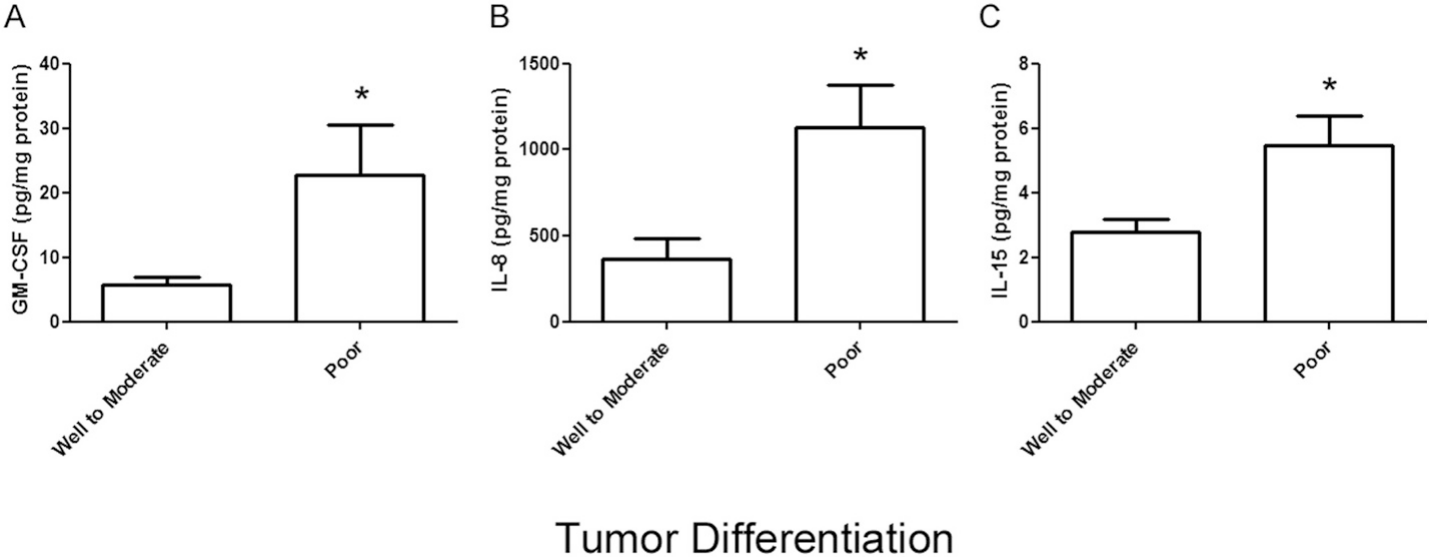
Inflammatory milieu within the tumor microenvironment correlates with tumor grade. Intratumoral concentrations of **a** GM-CSF, **b** IL-8 and **c** IL-15 demonstrated significant correlations with high tumor grade. *P < 0.05 for each association using the Mann Whitney U test

In order to determine whether patterns of soluble mediator concentrations could offer further insights into the biology of PC, the inflammatory milieu was evaluated with respect to commonly used clinicopathologic parameters, such as positive lymph node ratio, serum CA 19–9 concentrations, tumor grade and tumor size. In our analysis of lymphatic metastasis using positive lymph node ratio, elevated intratumoral EGF concentrations associated with a high degree of lymphatic metastasis (*ρ* = 0.332, *P* = .048), while high concentrations of IL-4 displayed the opposite trend, correlating with reduced lymphatic metastasis (*ρ* = −0.377; *P* = .023) (Table 3). Additionally, IFN-γ (*ρ* = 0.391; *P* = .022) and RANTES (*ρ* = 0.475; *P* = .005) demonstrated significant positive correlations with serum CA 19–9 levels. High levels of IL-8 and IP-10 associated with larger tumors (*ρ* = 0.336; *P* = .042 and *ρ* = 0.373; *P* = .023 with respect to tumor size in cm). The inflammatory milieu was then correlated with the degree of tumor differentiation observed in malignant tissue. Significant associations between poor tumor differentation and high concentrations of GM-CSF, IL-15 and IL-8 were observed (Fig. 2). While these patterns provide insights into potential relationships between aspects of clinicopathological parameters and inflammation, these parameters do not always correlate with outcome.

**Table 3:** Inflammatory milieu within the tumor microenvironment correlates with clinicopathologic parameters in PC specimens

Clinical parameter	Ligand	Spearman coefficient	*P* value
Positive Lymph Node Ratio	EGF	.332	.048
	IL-4	-.377	.023
CA 19–9 (U/mL)	IFN-ɤ	.391	.022
	RANTES	.475	.005
Tumor Size (cm)	IL-8	.336	.045
	IP-10	.357	.033

All significant comparisons are shown for which *P* < 0.05

We therefore aimed to determine if these known clinicopathologic predictors of outcome correlated with survival in our cohort. One commonly used predictor of overall survival in resected pancreatic cancer is the ratio of lymph nodes containing malignancy to the total number of lymph nodes resected and examined [31]. Indeed in our cohort, elevated positive lymph node ratios correlated strongly with reduced overall survival (HR 55.8; *P* = .002) (Table 4). This population therefore confirms the predictive nature of lymphatic metastasis. However, associations between survival and other commonly used prognostic parameters such as microscopically positive resection margins and poor tumor differentiation were not statistically significant in this cohort (HR 1.96; *P* = .12 and HR 2.28; *P* = .053, respectively) (Table 4).

**Table 4:** Univariate analysis of overall survival

	HR	95 % CI	*P* value
Age (y)	1.01	0.98–1.05	.556
Neoadjuvant Therapy	1.08	0.43–2.68	.870
CA 19–9 (kU/mL)	1.38	0.83–2.29	.215
Major Vascular Resection	2.66	0.85–8.30	.093
R1 Resection	1.96	0.84–4.53	.118
Positive Lymph Node Ratio	55.8	4.35–714	.002*
Poor Tumor Differentiation	2.28	0.99–5.23	.053
Tumor Size (cm)	1.06	0.89–1.28	.506

Clinicopathologic parameters were analyzed in a Cox proportional hazards model for 36 patients with surgically resected pancreatic adenocarcinoma. Abbreviations: *HR* hazard ratio, *CI* confidence interval, *y* years, R1 resection denotes a microscopically positive margin; Positive lymph node ratio refers to the number of lymph nodes positive for malignancy divided by the total number of lymph nodes examined; *cm* centimeters. *, *P* < .05

### Variations in the inflammatory milieu within the tumor microenvironment correlate with patient survival

**Fig. 3:**
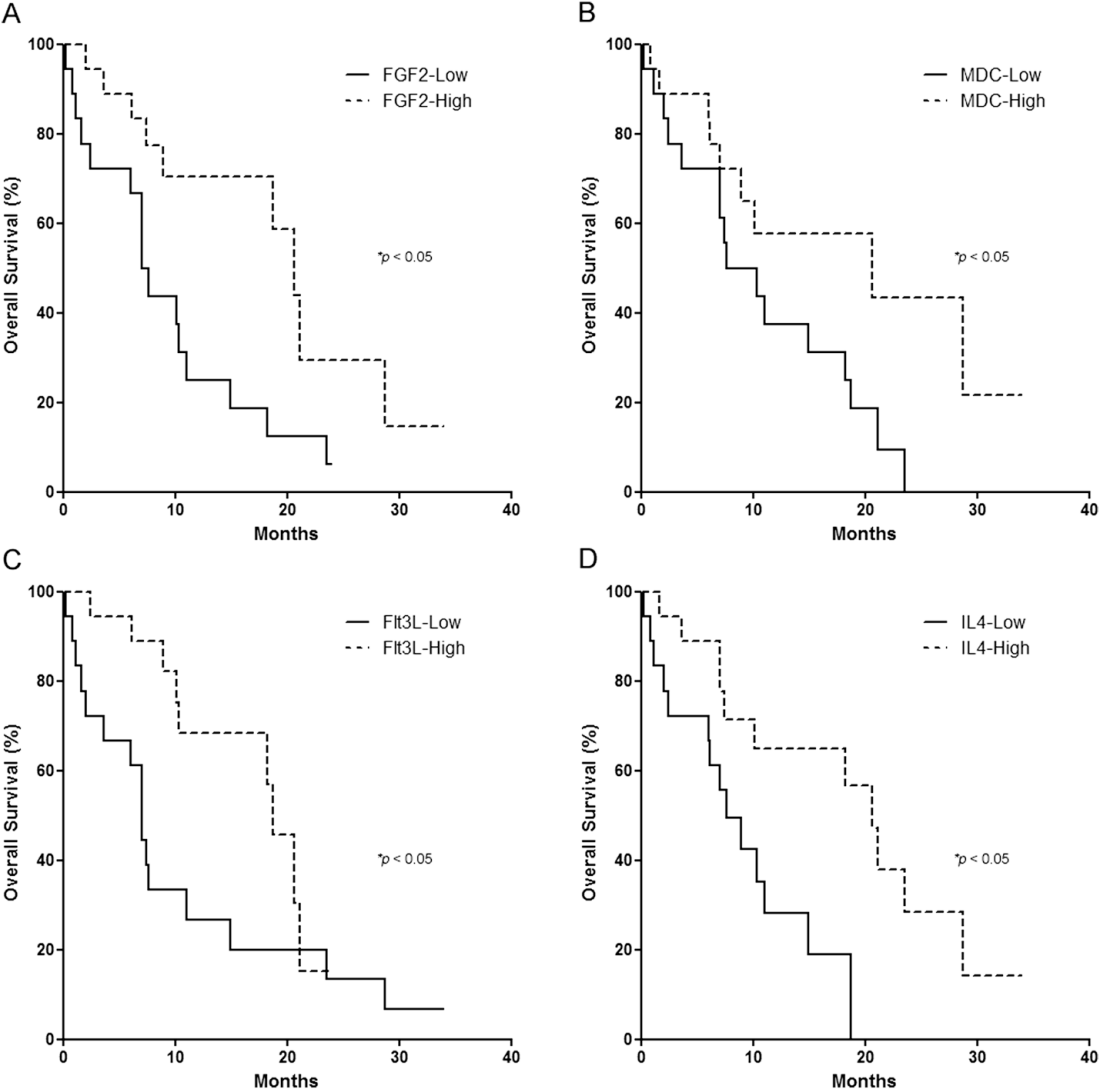
Elements of the inflammatory milieu within the tumor microenvironment have prognostic value. Kaplan-Meier curves are plotted for **a** FGF-2, **b** MDC, **c** Flt-3 L and **d** IL-4 based on cutoffs at median cytokine concentrations in 36 pancreatic adenocarcinoma specimens. The log-rank test was used to compare differences. Significance was considered for those in which *P* < 0.05

Since the common clinicopathological data were poor at predicting outcome, we next hypothesized that elements within the inflammatory milieu harbor superior prognostic value. The intratumoral milieu was correlated with overall survival in all 36 patients with PC who underwent surgical resection with curative intent. All patients had at least 6 months of clinical follow-up. Associations between the intratumoral milieu and overall survival were evaluated using both Kaplan-Meier and Cox proportional hazards models. Soluble mediators were first dichotomized using median concentrations and evaluated by log-rank test in a Kaplan-Meier model. FGF-2, MDC, IL-4 and Flt-3 L significantly correlated with prolonged survival upon dichotomization (Table 5, Fig. 3). Due to the potential bias introduced from the artificial categorization of values dichotomized at the median, a proportional hazards model was employed, allowing for direct correlation of intratumoral mediator concentration and survival. In this manner, high levels of both G-CSF and PDGF-AA correlated with reduced survival (HR 1.03; *P* = .016 and HR 3.51; *P* = .012, respectively) (Table 5).

**Table 5:** Soluble mediators detected within the PC microenvironment correlate with prognosis

	Dichotomized at median concentration			Univariate PH model		Multivariate PH model	
	Median OS (Low)	Median OS (High)	*P* value	HR (95 % CI)	*P* value	HR (95 % CI)	*P* value
EGF	10.4	11.0	.165	1.23 (0.98–1.54)	.078	1.10 (.86–1.42)	.439
G-CSF	18.3	7.5	.064	1.03 (1.01–1.06)	.016*	1.02 (1.00–1.05)	.071
PDGF-AA	18.7	8.9	.170	3.51 (1.32–9.31)	.012*	2.72 (1.00–7.40)	.050*
IL-6	11.0	10.4	.500	12.0 (0.85–172)	.066	6.10 (0.39–96.13)	.198
FGF-2	7.1	20.7	.010*	0.60 (0.37–0.97)	.038*	0.59 (0.37–0.93)	.024*
TNFα	7.5	18.3	.085	0.78 (0.63–0.98)	.031*	0.79 (0.64–0.97)	.027*
MIP-1α	10.4	14.9	.352	0.98 (0.96–1.00)	.036*	0.98 (0.97–1.00)	.029*
IL-4	7.6	20.7	.008*	0.85 (0.70–1.04)	.112	0.26 (0.75–1.08)	.902
Flt-3 L	7.1	18.7	.037*	0.95 (0.88–1.01)	.103	0.94 (0.86–1.02)	.113
MDC	7.6	20.7	.049*	0.01 (0–1.37)	.065	0.01 (0–2.24)	.098
Eotaxin	7.5	18.7	.055	0.03 (0–15.2)	.263	0.09 (0–47.9)	.454

Soluble mediators were dichotomized at median concentrations and survival was evaluated using Kaplan-Meier analysis with *P* values determined using the log-rank test (*left*). Associations between soluble mediator concentrations and survival were then evaluated in a continuous fashion using a Cox proportional hazards model (*middle*). Finally, concentrations were evaluated in a multivariate Cox proportional hazards model with positive lymph node ratio (*right*). Abbreviations: *PH* proportional hazards, *OS* overall survival, *HR* hazard ratio, *CI* confidence interval. *, *P* < .05

Anecdotal observations of interest included: Four patients with tumors which demonstrated distinctly high PDGF-AA concentrations, at least double that of any other PC specimen, recurred within 6 months postoperatively. Further, two patients with tumors containing G-CSF concentrations over 5 times that of any other also recurred within 6 months, with one of these patients showing evidence of metastatic disease as soon as two months postoperatively. Conversely, two patients with tumors with undetectable MIP-1α recurred within 6 months and three patients whose tumors had the highest intratumoral MIP-1α concentrations were recurrence-free between one and three years postoperatively. Further, the patient with the highest intratumoral TNFα concentration remains recurrence-free 34 months postoperatively.

## Discussion

Due to the dismal clinical outcomes associated with pancreatic adenocarcinoma and the continued debate surrounding therapeutic interventions, there is a tremendous need for the development of tools that can supplement current diagnostic and prognostic efforts. The extent of genetic and phenotypic heterogeneity specific to PC represents a major obstacle to the clinical application of developing biomarkers in PC. Efforts to further understand clinical observations regarding pathologic signaling within the tumor microenvironment have provided a novel focus that have led to major breakthroughs. Examples include therapeutic successes following Nab-paclitaxel infusion that is dependent upon SPARC expression in desmoplastic tumor-associated stroma [32, 33], and consistent observations that partial responses achieved from CD40 agonists led to the infiltration of tumoricidal macrophages into the local microenvironment [34, 35]. In order to further understand clinically important paracrine signaling pathways within this local microenvironment, the work presented here has detected a unique inflammatory signature within pancreatic adenocarcinoma that is distinct from that of chronic, benign inflammation. Further, several members of this panel of markers were associated with specific clinicopathologic parameters, response to cytotoxic chemotherapy, and overall survival.

The almost universal development of treatment resistance and disease relapse following systemic cytotoxic or targeted therapies has made survival in PC achievable in only a small minority of patients. Mechanistically, chemoresistant phenotypes have been reproduced in vitro. However, the relevance of these findings to clinical practice remains unclear. For example, gemcitabine resistance has been linked to the expression of gemcitabine-metabolizing proteins and DNA repair enzymes as well as the downregulation of nucleoside transporters. However, the clinical value of identifying these markers in resected PC specimens has yielded conflicting results [36]. Here we demonstrate that not only is the exposure to gemcitabine-based therapy associated with a different inflammatory milieu within the tumor, but also that differences in the milieu associate with the degree of clinical response, whereby increased levels of intratumoral IL1-β and TNF-α are associated with poor histopathologic response to neoadjuvant therapy. These findings further support a wealth of investigations linking downstream NF-κB signaling to tumor progression and chemoresistance [37].

The observation that EGF levels correlated with the degree of lymph node metastasis is consistent with widespread evidence implicating EGF signaling in cancer progression and metastasis, culminating in a phase three trial employing EGFR inhibition in pancreatic cancer [3]. Conversely, high intratumoral concentrations of IL-4 displayed the opposite trend, correlating with reduced lymphatic metastasis, whereby patients with tumors high in IL-4 concentrations displayed roughly triple the survival of those with tumors expressing low levels of IL-4. In light of this finding it is important to note that direct stimulation of cancer cells with IL-4 generally results in augmented growth and proliferation [38–40]. However, this finding must be interpreted within the context of IL-4 signaling within the microenvironment. Indeed, constitutive IL-4 expressing cancers have demonstrated reduced growth in vivo due to the induction of a robust antitumor immune response [41]. Similarly, intratumoral levels of IL-8 and GM-CSF were predictive of tumor grade while IL-8 levels were also positively associated with tumor size. Again this is consistent with previous findings that suggest that IL-8 and GM-CSF produced in the tumor microenvironment promote immune evasion in PC [42–44]. Interestingly, the administration of cytotoxic chemotherapy was strongly associated with significantly lower intratumoral IL-8 concentrations. The investigation of the intratumoral inflammatory milieu has therefore revealed consistent correlations between IL-8 concentrations, histopathologic findings and the administration of chemotherapy.

As alluded to above, several of these mediators could also be used to predict survival. For instance, high intratumoral G-CSF levels correlated with reduced overall survival, which is supported in literature relating myeloid-derived suppressor cell infiltration to tumor progression and angiogenesis [45]. Of particular interest is the prolonged survival observed in patients with high intratumoral FGF-2, known to stimulate fibroblast migration, wound healing and generally thought to be a growth factor which supports growing tumors. However, it is generally accepted that FGF-2 is abundant in most tissues, concentrated in basement membranes and at cell surfaces in inactive forms. Tissue injury leads to FGF-2 activation and subsequent promotion of wound healing processes known to promote tumor growth, invasion and angiogenesis [46]. In this context, reduced FGF-2 concentrations in tissue homogenates may paradoxically reflect increased FGF-2 activation, which would lead to the expected findings of reduced survival.

The inability to follow the intratumoral inflammatory milieu over time represents a significant limitation to this type of analysis, as this information will be critical to elucidating the relationship between local inflammation and treatment strategies in PC. In addition, stratification of long-term survival into treatment-naïve and treatment-exposed tumors will be essential in validating these relationships. However, this analysis currently lacks the power to dichotomize in this fashion. While grouping mediators into functionally relevant categories may address our current lack of power, the pleiotropic nature of these soluble mediators may lead to improper interpretations in the absence of functional analyses. Further, it has not escaped our notice that VEGF demonstrated no correlation with survival in this analysis. The extensive body of work associating VEGF signaling with angiogenesis and tumor progression has led many groups to investigate potential correlations between VEGF expression and survival in PC. Subsequent analyses have yielded conflicting results [47–49]. Importantly, this is not the first clinical cohort to demonstrate a nonsignificant correlation between intratumoral VEGF levels and overall survival in PC.

## Conclusions

In summary, pancreatic adenocarcinoma is a devastating malignancy with an extremely poor prognosis. High ratios of tumor stroma to cancer cells plague the sensitivity of cytologic diagnosis of PC; In fact, even direct pathologic analysis of PC biopsies can yield inconsistent results with high interobserver variability [50]. Highly specific, reliable biochemical signatures obtained from these small samples that improve diagnostic sensitivity could dramatically improve the clinical care of PC patients. Further, treatment algorithms for PC in the absence of metastasis are currently anatomic based and lack attention to variations in biology. Thus, there is further need to develop accurate biomarkers capable of predicting response to systemic therapies or the futility of surgical or radiation therapy. Unfortunately, the current literature is characterized by marked variability between individual studies as to the relative prognostic impact of several biomarkers in PC. Here, in contrast to studies that evaluated these properties using a single or a couple biomarkers, we have identified novel relationships between tissue examination and clinical outcome by quantitatively evaluating the milieu of the tumor microenvironment utilizing fresh pancreatic surgical specimens. It is important to note that even though some of these associations are counterintuitive based on the currently understood biology, the greater context and complexity of the tumor microenvironment in PC is not currently appreciated. Thus, these data combined with a better understanding of the context-dependence of inflammatory signaling, may eventually offer the opportunity to identify patterns that improve interpretations in cancer and emphasize the importance of investigating the tumor microenvironment as a whole. Nonetheless, results presented here exhibit a high degree of reproducibility and provide rationale to prospectively evaluate these markers as diagnostic and prognostic tools.
